# Tissue engineering in female pelvic floor reconstruction

**DOI:** 10.1002/elsc.202000003

**Published:** 2020-04-14

**Authors:** Xiaotong Wu, YuanYuan Jia, Xiuli Sun, Jianliu Wang

**Affiliations:** ^1^ Department of Obstetrics and Gynecology Peking University People's Hospital Beijing P. R. China; ^2^ Beijing Key Laboratory of Female Pelvic Floor Disorders Beijing P. R. China

**Keywords:** biological scaffold materials, pelvic floor dysfunction disease, seed cells, stem cells, tissue engineering

## Abstract

Pelvic organ prolapse is a common and frequently occurring disease in middle‐aged and elderly women. Mesh implantation is an ideal surgical treatment. The polypropylene mesh commonly used in clinical practice has good mechanical properties, but there are long‐term complications. The application of tissue engineering technology in the treatment of pelvic organ prolapse disease can not only meet the mechanical requirements of pelvic floor support, but also be more biocompatible than traditional polypropylene mesh, and can promote tissue repair to a certain extent. In this paper, the progress of tissue engineering was summarized to understand the application of tissue engineering in the treatment of pelvic organ prolapse disease and will help in research.

**Abbreviations**
PFD: pelvic floor dysfunction
POP: pelvic organ prolapse

## 1 INTRODUCTION

Pelvic organ prolapse (POP) is a common debilitating condition affecting about 30–40% of women. POP is the herniation of pelvic organs into the vagina with symptoms of bladder, bowel, and sexual dysfunction [1]. The POP etiology is multifactorial; ageing, obesity, pregnancy, parity, genetics, history of diabetes, and hypertension impact its progression [2]. Prevalence of POP varies in different geographical regions. The annual POP incidence in the USA is reported to be 31.8% over 2–8 years in a follow‐up study in menopausal women [3]. The rate of vault prolapse is reported to be between 4.4 and 6–8% in two European countries, Italy and Austria, respectively [4, 5] and the mean prevalence in developing countries is about 19.7% [6]. At present, the commonly used surgical methods in clinical practice include autologous tissue repair surgery and scaffold implantation surgery. The recurrence rate of autologous tissue repair is high, so mesh implantation becomes the focus of pelvic floor reconstruction. The purpose of using mesh in the treatment of pelvic floor dysfunction (PFD) disease is to replace autologous tissue with mesh and reconstruct the pelvic floor defect structure. According to the source of the materials, it can be divided into chemical synthetic mesh and biological mesh: synthetic mesh, such as polypropylene mesh, is the earliest applied in pelvic floor repair, with good mechanical properties and low recurrence rate. However, its non‐degradation and poor histocompatibility lead to a series of postoperative problems, such as graft erosion, exposure, and infection [7]. Biological mesh has good biocompatibility, but its mechanical properties are poor, the graft gradually degrade, and the degradation rate is faster than the formation rate of new tissue, resulting in decreased mechanical ability and recurrence of PFD, thus limiting its clinical promotion and application. Therefore, there is a void in the clinical management of POP that requires innovative cell‐based and tissue engineering approaches. Tissue engineering uses a combination of cells, biomaterials, growth factors and/or drugs implanted into an area of tissue damage or loss. The purpose of the cellular component is to accelerate repair and promote regeneration of damaged or lost tissue, while the material provides physical support and niches to deliver cells to the tissue. The first model used to demonstrate proof of principle of this approach for POP repair was in a rat skin wound repair model. It used a novel source of MSC from the endometrium with a newly designed knit material fabricated from FDA‐approved nondegradable polyamide dip‐coated in gelatin for MSC seeding and delivery [8]. We will summarize the research status of tissue engineering on female pelvic floor restoration.

## 2 SEED CELLS

Seed cells in tissue engineering is the prerequisite of tissue regeneration, including autograft, allograft, and heterogeneous groups, generally speaking, seed cells should meet the following conditions: a wide range of sources, some biological characteristics, and no immune rejection. Sources of seed cells for female pelvic floor repair include fibroblasts and stem cells.

### 2.1 Fibroblasts

Fibroblasts is a pelvic floor support structure, the extracellular matrix cells, can be drawn from the vaginal wall, ligament structure, etc., and it can secrete collagen, elastin, cytokines, etc. Jia et al. [9] found that fibroblasts were significantly increased in growth rate and collagen expression by stimulating them in vitro. Fibroblasts were planted on polypropylene mesh and some biological meshes, and a collagen membrane formed by extracellular matrix secreted by fibroblasts at the implant site and between the meshes was used to improve the biocompatibility of the mesh and reduce complications [10, 11, 12]. Drewa et al. [13] successfully repaired abdominal wall defects of mice by planting cultured fibroblasts on polyglycolic acid (PGA) scaffolds, suggesting that fibroblasts in tissue engineering can synthetic extracellular matrix to achieve the goal of restoration, but women with PFD, a large number of studies have also shown that patients collagen metabolism are disorder, so autologous fascia of the patients compared with the same intensity and the recurrence rate requires authentication. A 10‐year follow‐up study [14] showed that the long‐term effects of TVT sling and autologous fascia graft were the same, and in fact, autologous fascia graft had higher strength than TVT sling. Obviously, the autogenous fibroblast is a good seed cell.

### 2.2 Stem cells

Recent advances in stem cell‐based therapeutics have propelled an increasingly high enthusiasm in tissue engineering. The essential ingredients for successful tissue engineering include the choice of biomaterials combined with the appropriate cells and growth‐inducing factors [15].

#### 2.2.1 Embryonic stem cells

Different kinds of stem cells have different functions. Embryonic stem cells, usually derived from embryos or fetuses, can differentiate into any type of cell or germ layer. Human embryonic stem cells (hESC) are derived from blastocysts and possess a high degree of plasticity and rapid proliferation. Theoretically, embryonic stem cells can differentiate into any cell. However, it is technically strict to induce the targeted differentiation of embryonic stem cells to achieve the purpose of transplantation and reduce tumorigenicity [16]. In addition, the immunogenicity after embryonic stem cell transplantation needs to be emphasized. These cell lines acquire human leukocyte antigen (HLA) when they mature, so patients transplant tissue engineering products from embryonic stem cells as seed cells may need immunosuppressive therapy [17]. On the other hand, the ethical issues involved in embryonic stem cell transplantation also limit its application in the treatment of pelvic floor diseases. Pluripotent stem cells, including endometrial mesenchymal stem cells(eMSC), mesenchymal stem cells (MSC) and adipose stem cells (ADSC) from the blood, bone marrow, placenta, or fat tissue, have been an interest for allogeneic cell‐based therapies for decades [18].

**PRACTICAL APPLICATION**

Female pelvic floor repair polypropylene mesh has been banned in 2019 after the FDA warned of complications such as erosion and exposure. Therefore, tissue engineering technology for female pelvic floor repair came into being. However, the application of tissue engineering in the field of female pelvic floor is rarely reviewed. In this work, we review the application of tissue engineering in the field female of pelvic floor, hoping to provide theory for guiding the treatment of pelvic floor dysfunction.

#### 2.2.2 Induced pluripotent stem cells

In 2006, potentialization and maintaining pluripotency viral candidate cells into the mouse skin after screening, are finally Oct4, Sox‐2, c‐Myc, and Klf4 can be introduced into the four transcription factor genes. To fiberize mature rat skin dimensional cell reprogramming into embryonic stem embryonic stem cells (ESCs)‐like cells, called inducible pluripotent stem cells (iPSCs) [19] .hiPSC has the ability of self‐renewal, which refers to the “infinite” proliferation ability that is produced in the mother's cells by the symmetrical division, and can also be called “immortality”; it has the potential of multidirectional differentiation can differentiate into mature cells with different phenotypes. hiPSC have a complete genome, that is, they contain all the genetic information of the source cells. Compared with other stem cells, hiPSC are derived from autologous cells or other types of cells, so it can avoid immune rejection caused by allogeneic transplantation; in addition, it does not need to be taken from the cell mass in the early embryo of mammals, which prevents embryo ethical controversy caused by stem cells. hiPSC can be differentiated into embryoid bodies containing various germ layer sources under appropriate stimulation, and can be induced into disease‐related functional cells, such as insulin‐secreting cells, hematopoietic cells, nerve cells, etc. hiPSC technology is mainly simulated from the genetic aspects of the human body, so it is difficult to construct an ideal in vitro cell model and perform cell therapy for diseases with environmental factors as the leading role. The mechanism of differentiation after transplantation of hiPSC into the body is not clear. Autologous transplantation of induced pluripotent stem cells has been reported to be immunogenic [20]. Currently, induced pluripotent stem cell technology can be used to induce cells to differentiate into desired targets in vitro. However, little is known about the differentiation mechanism of the differentiated cells after transplanted into the body. Therefore, the mechanism of induced differentiation of induced pluripotent stem cells needs to be further studied. In addition, there is currently no specific detection system to evaluate the efficiency and safety of functional cells after transplantation. Although hiPSC technology increases the source of stem cells, it also increases the risk of cell mutations, which affects the safety of hiPSC. Nowadays, the research on the technical mechanism of induced pluripotent stem cells has gradually shifted from the level of transcription factor‐related transcriptome and proteome to epigenetics. The variation induced by classical methods induced by pluripotent stem cells mainly comes from chromosomal abnormalities and gene copies There are several aspects such as number mutation and point mutation. Studies have shown that Brg1 and BAF155 in the embryonic stem cell‐specific chromatin remodeling complex BAF can coordinate the reprogramming of fibroblasts with the three factors Oct4, Sox2, and Klf4. These chromatin remodeling is not only conducive to Oct4 gene regulation, on the other hand, also enhances the ability of the Oct4 protein to bind to the promoter regions of Sall4, Tcf3, and Dppa4 genes. Some mutations also occur during the induction process, and some somatic cells are damaged during the induction process. The induction itself also causes an increase in copy mutations, and the longer the culture time, the more the accumulation of mutations. In addition, different detection methods have different results for the genetic stability of genetic analysis techniques have found more genetic abnormal hiPSC. Modern molecular abilities of hiPSC than traditional cytogenetic analysis techniques. The potential carcinogenicity of hiPSC factors has increased people's concerns about the safety of clinical application of hiPSC technology to a certain extent, but it has also stimulated in‐depth research on inducible factors by researchers. It has been reported that >40% of genes that exhibit mutations at the level of genetic mutations are associated with tumors [21]. The study found that the six induction factors (Oct3/4, Sox2, c‐Myc, Klf4, Lin28, Nanog) used in induced pluripotent stem cell technology, except Lin28 has not been found to be related to tumorigenesis, the remaining five are oncogenes, whose overexpression is often associated with tumors [22]. C‐Myc is a proto‐oncogene [23] that can be detected in a variety of tumors and can promote cell proliferation and transformation [24]. Nakagawa studied the combination of Oct4, Sox2, and Klf4 and found that the lack of c‐Myc can lead to its cell induction disorder, its tumorigenicity inhibits reprogramming, and it also increases the frequency of cell transitions during the passage of hiPSC. When Myc transgene continues to play a role in hiPSC, it can also increase the risk of tumor formation. Oct3/4 can induce cells to change into embryonic stem cells away from tumor cells, and its forced expression can maintain the morphology of embryonic stem cells [25]. In recent years, the research of hiPSC has attracted much attention from the scientific and medical communities, and has worked hard to overcome the problems of immunity and ethics, and successfully obtained induced hiPSC. However, due to the inability to solve the safety, efficiency, and mechanism of hiPSC differentiation in the technology, it is limited to theoretical and laboratory research, and it has not been applied to the clinic. How to safely and efficiently induce hiPSC into the type required by patients and transplant them, how to establish a good disease model, and establish a high‐throughput drug screening platform involve basic research in all aspects, facing huge difficulties and challenges.

#### 2.2.3 Endometrial mesenchymal stem cells

A rare type of perivascular mesenchymal stem cells found in the endometrium, which can be easily obtained from endometrial biopsy or even postmenopausal women [26, 27]. Ulrich et al. have demonstrated in phase IV clinical trials that postmenopausal women can regenerate endometrial tissue [28]. Many clinical trials of MSC therapies exploit their anti‐inflammatory and immunomodulatory properties. eMSC also have significant immunomodulatory function, influencing macrophage switching from M1 inflammatory to a M2 wound healing phenotype in rodent models used to assess new biomaterials for treating POP [29, 30]. The M2/M1 ratio is crucial for the success of implanted mesh. eMSC have beneficial effects on subcutaneously implanted nondegradable eMSC/PA/G tissue engineering constructs, reducing the release of host macrophage inflammatory cytokines, TNF‐α and IL‐1b in both C57BL6 and immunocompromised NSG mice. eMSCs can be a good choice for seed cells.

#### 2.2.4 Skeletal muscle stem cells

Ho et al. [31] based on cell therapy for pelvic floor dysfunction, they were used in vitro culture of mice skeletal muscle stem cells (MDSC) that seeded on decellularized matrix from pig small intestinal submucosa (SIS) implanted in rats vagina, MDSC differentiated into smooth muscle cells, and promote the vaginal tissue repair. Boennelycke et al. [32] seeded fresh muscle fiber fragments on biodegradable PLGA scaffolds, constructed tissue engineering meshes, and implanted them under the abdominal skin of rats. After 8 weeks, new muscle fibers grew and the scaffolds were degraded. Muscle satellite cells on newly isolated muscle fibers may be the key cells responsible for tissue regeneration, whether it can replace myogenic stem cells in the treatment of POP is still controversial, but in animal studies, isolated autologous muscle tissue or muscle fragments have been used in the treatment of SUI [33] and abdominal hernia [34]. In the treatment of female urinary disease, researchers and clinicians have been trying to use injection to induce urethral sphincter muscle regeneration, in vivo study showed that cells can survive a period after injection, reconstruction's process is similar to normal skeletal muscle regeneration process, new nerve fibers, smooth muscle cells, loose stromal tissue, and blood vessels formed. MDSC can be a good candidate for POP.

#### 2.2.5 Mesenchymal stem cells

Easy to be isolated, cultured, and amplified from bone marrow or adipose tissue, have been widely used in the repair and regeneration of damaged tissues [35, 36]. In gynecological and urologic diseases, bone marrow stromal stem cells (BMSC) and adipose stromal stem cells (ADSC) were used in animal models to repair urethral sphincter for SUI [37, 38]. Zou et al. [39] successfully improved the symptoms of SUI in rats by implanting BMSC. Dolce et al. [40] demonstrated that BMSC grew well on PGA mesh and reduced the degree of abdominal adhesion in rats, improving the biocompatibility of the mesh. MSC are pluripotent and can be differentiated into different lineages such as bone, cartilage, fat cells, tendons, ligaments, and smooth muscle. The direction of differentiation is driven by the microenvironment of the implant site. In this way, autologous MSC, especially the readily available ADSC, may be an ideal candidate for POP repair. To date, nearly 500 clinical trials using mesenchymal stem cells (MSC) have treated more than 2000 patients [27]. They were use autologous or allogeneic mesenchymal stem cells as cell suspensions to inject. many involve intravenous inject. So far, despite there were many reasonable preclinical evidence, the therapeutic effect of these trials is marginal. Consensus on the therapeutic mechanism of mesenchymal stem cells does not exist yet. Nevertheless, there are some hypotheses to explain the observed clinical benefits of MSC [41], (1) the intrinsic ability to differentiate into different cell lineages, (2) producing an array of soluble bioactive factors for cell maintenance, survival, and proliferation, and (3) regulating immune responses and (4) migrating recruitment site to alleviate injury and promote reconstruct [42]. In some reported cases, MSC seems to avoid allograft rejection in both human and animal models [43, 44, 45, 46, 47]. More practically, the allogeneic cell source must be able to demonstrate its reliable ability to elicit meaningful therapy under the immune capacity for patient allogeneic tissue, which includes reliable cell homing and partial dose accumulation or retention at sites of interest for sufficient time to complete reconstruct [48]. It is currently estimated that less than 3% of injected stem cells remain in the injured myocardium 3 days after the ischemic injury injection [49]. In addition, most cells transplanted into the target tissue will die within the first few weeks. The effective transformation of mesenchymal stem cell therapy is currently hindered by the clinical inability to target these therapeutic cells to specific tissues with reasonable efficiency and significant transplantation and retention [50]. Traditional MSC therapies are inject cell suspensions derived from adherent cells obtained from cultured plastics using proteolytic enzymes. Proteolytic free cells take a long time to recover from harvesting, suspension, and loss of cell connections, associated matrices, and cell receptors. MSC maintained in a 2D culture system has been shown to gradually lose its inherent proliferation potential, colony formation efficiency, and differentiation ability with passage [51, 52, 53]. In addition, as proteolytic enzyme treatment destroys the adhesion components and mechanism of endogenous mesenchymal stem cells, homing to the target tissue region is impaired [54, 55]. Integrating healing physiology and regenerative potential are key factors in reducing low cell retention and embedding into target tissues and organs for a successful cell therapy [56].

### 2.3 The security of stem cells

In a recent study, ADSCs and their exosomes separated from cancer patients were safe and had therapeutic benefits, suggesting that expanded ADSCs donated by cancer patients were not affected by patients' conditions, including cancer [57]. Although ADSCs are precursors to many cell types, their vital important function is chemical signal and induce differentiation into specialized cells, including dermal fibroblasts and keratinocytes [58]. Fat cells themselves derived from ADSCs were successfully injected subcutaneously to treat soft tissue diseases [59, 60]. In one study, treated patients with ADSC and followed for one year without adverse events [61]. Although total fat tissue transplantation has been successfully used to treat finger ulcers in patients with systemic sclerosis, a 10 years of tumor follow‐up after fat transplantation showed no increased cancer risk in patients [62]. A meta‐analysis of 1453 fat transplant patients with mean follow‐up of 16.3 months (range:1–56 months) for breast reconstruction showed no increased incidence of breast cancer [63]. In vitro and in vivo studies have shown that MSCs can inhibit tumor growth, which is expected since MSCs can build ECM, while normal ECM inhibits tumor growth through a process of dynamic reciprocity [64]. In vitro studies have shown that in human cells, adipose tissue rather than fat‐derived stem cells can significantly increase the growth rate of breast cancer xenograft tumors [65]. In vitro, studies on 3D culture of ADSCs further demonstrated their safety in breast tissue, because under the stimulation of breast epithelial cell line HL‐100 [66]. ADSCs formed a structure similar to acinar and showed the characteristics of epithelial differentiation [67]. The relatively safety characteristics of ADSCs, including their nuclear stability as they proliferate, make these cells a valuable tool for cancer therapy [68], including the provision of paclitaxel to cancer patients [69]. Patients with an average age of about 50 years received autologous ADSCs injection for osteoarthritis and were followed up for 1 year, with no adverse events, improved pain degree, and reduced disease progression [70]. In a phase II trial, patients were followed up for 6 months after injection of allogeneic ADSCs for perianal fistula, most patients did not observe related adverse events, and the fistula was closed [71]. In another phase II trial, patients were followed up for 2 years after the injection of ADSCs allograft to treat Chron fistula, with no adverse reactions, and 80% of patients healed completely [72]. In phase IIb double‐blind, randomized, placebo‐controlled studies of ADSCs injection into osteoarthritis, no significant improvement, or adverse events were observed during the 6‐month follow‐up [73]. Therefore, the safety of ADSCs and their secretors has been well confirmed in humans even when injected [74].

### 2.4 The low immunogenicity of stem cells

ADSCs have also been shown to modulate immune function in a number of beneficial ways, rejecting the ability of ADSCs secretory bodies to improve the survival rate of skin allografts. A large number of studies have shown that ADSCs can inhibit the proliferation of activated T cells regardless of direct contact with ADSC‐T cells [75], while ADSCs secretory alone can inhibit the proliferation, differentiation, and activation of T cells [76]. Co‐culture of peripheral blood mononuclear cells and ADSCs can inhibit pro‐inflammatory T cells and induce it with regulatory phenotype and anti‐inflammatory response characteristics [77, 78]. Mesenchymal stem cells have low immunogenicity and are often used in allogeneic applications without immunosuppression. However, recent studies have shown that subsequent doses of mesenchymal stem cells can be more rapidly removed from the body. One advantage of autologous mesenchymal stem cells is their inherent compatibility with host tissue, allowing repeated administration. In the current study, to our knowledge, we have shown that autogenous eMSC last the longest, with about 6% surviving 30 days in vivo. The longer mesenchymal stem cells remain in the body, the greater their role may be, especially in repairing damaged tissues or regulating the response of allografts or meshes [79].

## 3 BIOLOGICAL SCAFFOLDS

In nature, cell behavior and tissue structural development occur within the nanoscale architecture of the extracellular matrix (ECM). Vaginal wall anatomy shows that simple method of stem cell injection therapy to repair damaged tissue is not feasible, for most mammalian cells need adhesive matrix, researchers are turning to biological scaffolds with ECM‐like topography that more closely represent the vaginal ECM. Biological scaffold materials provide such a 3D matrix structure in which cells can adhere, proliferate, and differentiate with high efficiency. Tissue engineering biological scaffold materials should meet the following conditions [80]: (1) good biocompatibility, benefit to cell adhesion, proliferation, no toxicity, no immunogenicity; (2) biodegradable; (3) has a certain mechanical strength and guide tissue regeneration; and (4) a certain porosity and the appropriate size of the aperture. Tissue engineering biological scaffold materials used in pelvic floor dysfunction diseases include the following categories: synthetic materials and natural materials.

### 3.1 Synthetic materials

Including synthetic polymers. The most widely used synthetic polymers in tissue engineering are polyhydroxy acids [81, 82], including polylactic acid (PLA), polyglycolic acid (PGA) and its copolymer (PLGA). These synthetic polymers are naturally non‐toxic, and the degradation reaction is chemical hydrolysis. The products are lactic acid and glycolic acid, which are cleared by metabolism of the body [83]. Since its degradation is not dependent on the enzyme concentration in the local tissue environment, its degradation in vivo is controllable [84]. By changing the ratio and polymerization parameters of PLA, the properties of the synthesized polymer, such as tensile strength, young's modulus and degradation rate, can greatly meet the requirements of tissue engineering. In fact, these materials have been successfully applied in urethral tissue formation and bladder replacement [85, 86, 87]. In addition, hydrogels based on synthetic polymers can continuously release bioactive factors into tissues to regulate the differentiation of stem cells implanted on 3D polymer scaffolds [88]. Polycaprolactone (PCL) is another synthetic polymer based on hydroxyalkanic acid, which has been approved by FDA for clinical due to its excellent biocompatibility, low immunogenicity, hydrolysis under physiological conditions, and other excellent properties, and has attracted much attention in tissue engineering [89, 90]. Although the synthesis of polymeric biomaterials has the excellent properties of biological scaffold, but some kinds of polymers, such as poly hydroxy esters, may produce acid degradation products, this change in pH can affect the behavior of the cells and survival [91], and cause of the abnormal tissue and inflammatory response [92]. In addition, due to a lack of biological function domain, synthetic polymer itself are usually do not have immunogenicity, but at the same time, it cannot make the cell adhesion. Today's various synthetic technologies have optimize the synthetic polymer, the biological activity area coupling to the scaffold, so that they can produce bioactive bionic scaffold, for example, serum coating containing collagen or synthetic polymer scaffolds can make cells and extracellular matrix (ECM) deposition [93, 94]. In other cases, synthetic polymer scaffolds are prepared and modified by covalently fixing extracellular matrixderived molecular fragments to promote cell adhesion and enhance directed differentiation of stem cells [95]. In addition, the addition of biological activators on the surface of synthetic polymer scaffolds is one of the most effective ways to induce cell‐ECM‐like‐material interaction [96]. Biodegradable polymer scaffolds with functional groups were established on the surface of the material to initiate the required cell‐material interaction [97]. Ideally, an excellent cell scaffold, not only causing immune rejection, but have both excellent mechanical and biological properties: it contains a variety of tissues and cytokines, and provides an appropriate microenvironment to promote tissue regeneration [98]. This kind of material chemistry has exerted a fundamental and increasingly important influence on material science. In fact, the shape, size, structure, mechanics, porosity, surface texture, and other physical properties of scaffolds, once placed in the body's cellular microenvironment, will have a profound impact on the biological functions of biomaterials.

### 3.2 Natural materials

Natural biomaterials can generally be divided into two categories: protein biomaterials (such as collagen, silk fibroin, gelatin, fibonectin, keratin, etc.) and polysaccharide biomaterials (such as hyaluronic acid, cellulose, glucose, alginate, chondroitin, chitin and its derivatives chitosan, etc.). Protein‐based biomaterials are usually acquired from animal and human, including bioactive molecules that mimic the extracellular environment, whereas polysaccharide‐based biomaterials are mainly derived from algae, such as AGAR and alginate, or from microbial sources, such as dextran and its derivatives [99, 100]. Another type of natural biomaterials is tissue‐derived biomaterials, such as acellular matrix mesh, which is a new type of allogeneic biomaterials. Cell adhesion is mediated by specific integrin ligand interactions between the cell and its surrounding ECMs [101], so, the extracellular matrix of the basement membrane and dermis is an excellent biological scaffold, removing the immunogenicity while retaining the complete structure for cell adhesion and proliferation. The disadvantage is the lack of mechanical strength and degradation rate, resulting in the recurrence of prolapse [102]. To overcome these limitations, tissue engineering scaffolds have recently been redesigned and manufactured to mimic natural ECM ligands. These scaffolds are commonly used in vitro as natural ECM analogues to promote cell‐ECM interaction [103, 104]. For many years, it has been recognized that the quality of decellularization process is directly related to the immune response after scaffold implantation, so researchers have optimized the decellularization process to completely remove cell components and retain bioactive factors while maintaining ECM integrity [105, 106]. In the past few decades, there has been a great deal of research on biological information and components of natural ECM in biomaterial design. Based on its space mode, chemical composition and function, ECM components can usually be divided into two categories: the basement membrane (BM) and the stromal matrix (SM). The basilar membrane contains type IV collagen, laminin, basilar membrane glycan, aggregates, nesters, and other macromolecules that play a key organizational role in providing a membranous matrix for tissue's peripheral cells, including wrapping blood vessels as support for epithelial cells and maintaining cell polarity [107]. The matrix is composed of larger fibrous structures, which are the main structural of ECM. Although without cell components, ECM have various cytokines and biological signaling molecules, once implanted into the weak of pelvic floor tissue as tissue engineering products, these bioactive substances are released and play a natural role in cell regulation, thus providing the information necessary for repair and regeneration of specific ECM to guide cell growth, proliferation and differentiation [108]. Therefore, ECM as a “ready‐made” and immune‐compatible biomaterial has attracted more and more attention in tissue engineering (Table 1).

**Table 1:** Summary of materials commonly used in female pelvic floor tissue engineering

Materials	Application	Advantages
PLA	Urogynecological alternative implant in vitro study [109], ascorbic‐acid releasing biomaterials for pelvic floor repair [110,1112]	Phenotypical morphology and functionality maintained, increased cell metabolic activity and proliferation, matrix deposition, and collagen production [111]
PLGA/PCL	Implant for pelvic floor [112]	Comparative strength to native tissue, cell adhesion and growth, migration through the scaffold [112]
SILK	Urethra reconstruction [113],Tissue engineering mesh for pelvic floor reconstruction in abdominal wall rat model [114], biocompatibility, no inflammation, tissue growth [113], tissue ingrowth, degradation [114]	Cell migration, adhesion and proliferation [113], endothelial and smooth muscle cell attachment, and proliferation [115]
Collagen (type I, II, and III), cellulose	Tissue engineering scaffold [116, 117], urinary bladder regeneration [118], mesh for pelvic floor reconstruction in abdominal wall rat model [114]	Providing microstructure and cell adhesion, cell attach and proliferation [116, 117], cell penetration through implant, proliferation, matrix deposition [118]
BM/SM	Urinary bladder regeneration [118], mesh for pelvic floor reconstruction in abdominal wall rat model [114]	Natural ECM analogues to promote cell‐ECM interaction [105, 106].

#### 3.2.1 Foreign body reaction (FBR)

FBR is the physical reaction of biomaterials to foreign bodies after implantation, which is triggered by protein adsorption and eventually leads to excessive deposition of collagen around foreign bodies, resulting fibrous [119]. The physical and chemical properties, size, morphology, chemical properties and degradation rate of biological materials determine the final result of FBR [119]. When the scaffold is in contact with the extracellular matrix (ECM), the FBR process begins, causing the complement and the inherent coagulation system to activate and imminently adsorb blood proteins (albumin, fibronectin, fibrinogen, complement protein, and globulin) to the graft surface. A matrix is formed around the biomaterial prior to interaction with host cells [120]. These adsorbed proteins regulate the host cell response and the overall immune response, leading to the formation of a temporary matrix, usually a thrombus (blood clot) at the interface between the material and the host tissue [120]. These proteins, including a rich and effective mixture of cytokines, chemokines, growth factors, and cell‐secreted components, produce an environment that attracts inflammatory cells into the implantation site [121]. They also provide a structural and biochemical basis for the wound healing process and regulate subsequent FBR. MSC‐based biomaterial implants have been widely used in areas other than pelvic floor disease and tissue regeneration, with similar results including improved angiogenesis, M2 macrophage response, and reduced fibrosis [122]. The mechanism of MSC interaction with inflammatory cells has been investigated in a rat myocardial infarction and reperfusion model using a poly(ethylene)glycol hydrogel to promote repair [123].

## 4 BIOACTIVE FACTORS

Seed cells, biological scaffold materials and bioactive factors are also regarded as major elements of tissue engineering in modern view. The therapeutic effect of engineering mesh on pelvic floor dysfunction disease may also benefit from the addition of bioactive molecules in the scaffold. These bioactive factors may induce differentiation and enhance the regeneration process by activating stem cells [124, 125]. Pelvic floor dysfunction related factors include: estrogen, growth factors, growth factors such as basic fibroblast growth factor (bFGF), epidermal growth factor (EGF), transformed growth factor‐beta (TGF‐β), connective tissue growth factor (CTGF) and so on. The decellularized matrix mesh retains a variety of biological factors: bFGF, TGF‐β, and some polysaccharide and other extracellular matrix molecules to enhance the growth of cells and tissues at the implantation site [126, 127]. Studies have shown that estrogen promotes pelvic floor tissue repair by affecting fibroblast proliferation and collagen synthesis [128]. However, the therapeutic effect of estrogen is controversial. Takacs et al [129] showed that estrogen and selective estrogen receptor regulator levoxifen promoted the growth of vaginal smooth muscle cells in vitro, but inhibited the production of elastin. In the rat model, Manodoro et al. [130] found that estrogen increased the ability of the mesh to repair, but reduced the tensile strength of the implanted tissue. Hildebrand et al. [131] demonstrated that bFGF significantly promoted the differentiation of BMSCs into fibroblasts, and significantly increased the expression of ligament and tendon‐specific extracellular matrix and cytoskeletal components. Jia et al. [9] compared the effects of different concentrations of EGF and FGF on fibroblasts, and found that both growth and proliferation of cells and collagen expression were increased, suggesting that repair and regeneration could be promoted by changing the growth microenvironment of pelvic floor tissues. TGF‐β1 stimulated the secretory formation of the extracellular matrix and inhibited its degradation, stimulating the enhancement of mRNA levels of most collagen genes in cells and extracellular matrix and the increase in protein production. Connective tissue growth factor (CTGF), as a downstream signal of TGF‐β1, is a newly discovered growth factor that can stimulate the proliferation of fibroblasts and collagen deposition, and can promote mitosis, proliferation, chemotaxis, migration, and differentiation of fibroblasts [132].

## 5 CONSTRUCTION

**Figure 1:**
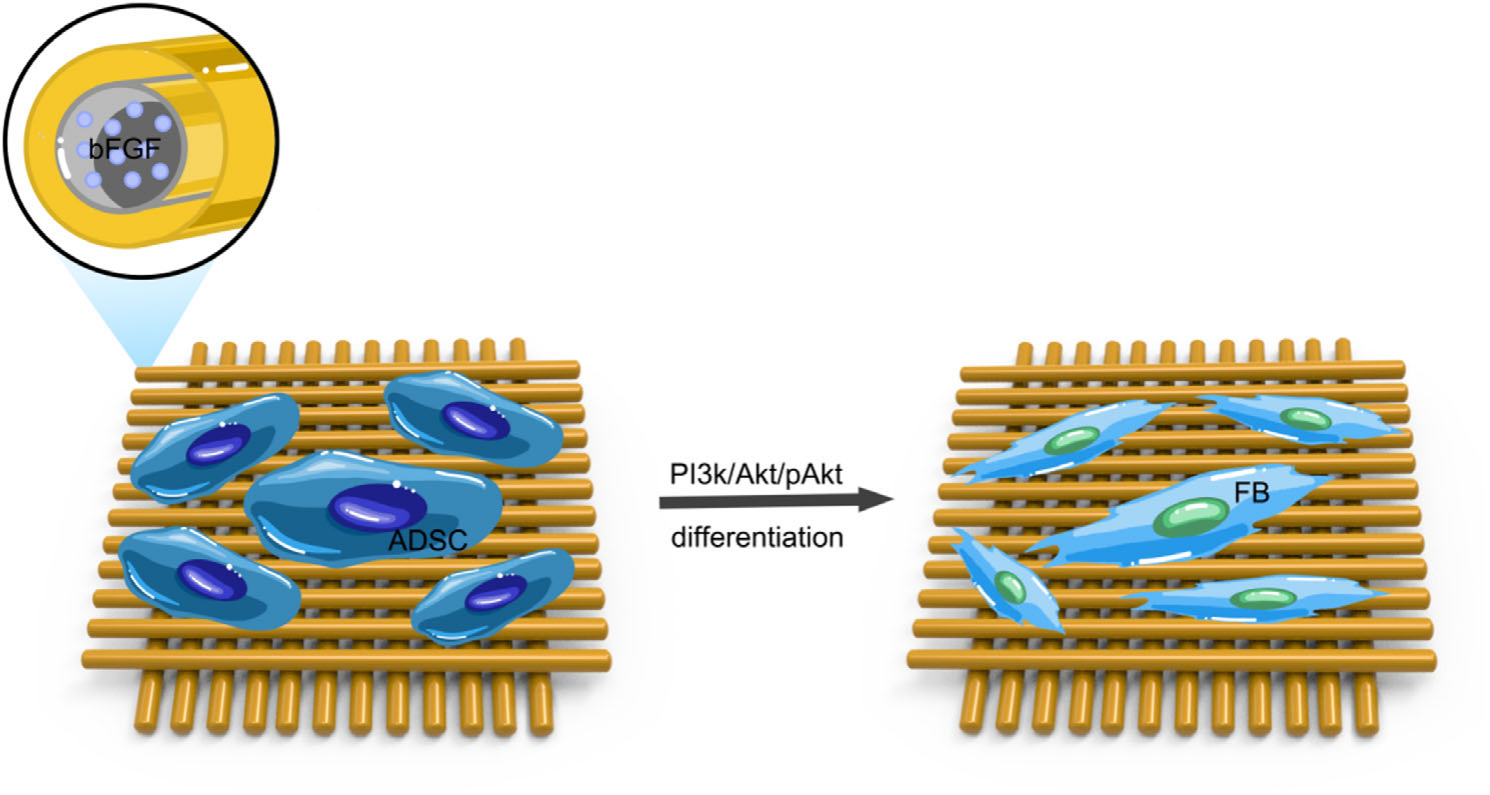
The simulation of tissue engineering mesh transplant into pelvic promotes stem cell differentiation into target cell

Tissue construction techniques are divided into in vivo construction and in vitro construction. In vitro construction: the seed cell with biological scaffolds in vitro environment culture, using the biological characteristics of scaffold and surface active factor, promoting the cell adhesion and secretion of extracellular matrix, the new extracellular matrix gradually accumulation complete the tissue repair, this method is easy to control, but the period is long, cells grow not divide evenly, poor mechanical strength in the new tissues in vitro, lead to failure or recurrence. In vivo construction: the cultured seed cells and scaffolds directly transplant into the body with short period, controllable scaffold strength, and no special culture conditions, which is a commonly used construction method, but the therapeutic effect is affected by the microenvironment of the implantation site (Figure 1) . The emergence of 3D biological printing technology to support production and the distribution of the cells have a very good control effect, is hot in tissue engineering of the emerging research method, compared with other methods, high precision, good cost‐effective, easy and feasible, and cell distribution good controllability [133, 134], including extrusion, inkjet, stereo lithographic method, laser‐assisted biological printing method, etc., successfully to seed the cells on scaffold.

## 6 PROSPECT

Mesh implantation is the mainstream surgical method for pelvic floor reconstruction. In view of the deficiency of chemical synthetic mesh and biological mesh, the construction of tissue engineering pelvic floor repair scaffold came into being. The regeneration of pelvic floor tissue was promoted by seed cells and bioactive factors, and the scaffold structure had a certain supporting capacity in the process of tissue repair. A growing body of evidence indicates that tissue engineering in the field of life science has made great achievement, along with the advance of synthetic technology and biological science, from the new cognition of biological systems and the new structure of human biological material, chemical, and physical insights into ceaselessly, the future will have a new, more complex tissue engineering design and inspiration. The impact of this area will continue to grow and develop as joint laboratories develop tissue engineering products that provide simple solutions to complex problems.

## CONFLICT OF INTEREST

The authors declare that they have no conflicts of interest.
